# lncRNA LINC01296 Promotes Oral Squamous Cell Carcinoma Development by Binding with SRSF1

**DOI:** 10.1155/2021/6661520

**Published:** 2021-06-11

**Authors:** Yanhui Zhang, Aifang Wang, Xiaohe Zhang, Xiaoliang Wang, Jin Zhang, Jinji Ma

**Affiliations:** ^1^Department of Stomatology, Jinan Central Hospital Affiliated to Shandong University, Jinan, China; ^2^Department of Stomatology, Jinan Eighth People's Hospital, Jinan, China; ^3^Department of Oral Disease Gaoxin Branch, Jinan Stomatological Hospital, Jinan, China

## Abstract

**Objective:**

Oral squamous cell carcinoma (OSCC) is the most common malignant tumor of the head and neck, with strong local invasiveness and cervical lymph node metastasis. The purpose of this study was to investigate the role of LINC01296 in oral squamous cell carcinoma and its possible mechanism.

**Materials and Methods:**

GEPAI database analysis and clinical samples were used to detect the expression of LINC01296 in head and neck cancer. In vivo experiment, MTT, clone formation assay, and transwell were used to detect the proliferation, migration, and invasion of oral squamous cell carcinoma. The effect of LINC01296 on EMT was detected by western blot and qRT-PCR to measure the expression of epithelial and mesenchymal phenotypic markers. BALB/c nude mice were used to carry out in vitro treatment experiment. In terms of mechanism, the binding relationship between LINC01296 and SRSF1 was predicted and verified by the RBPDB database and RNA pull-down assay.

**Results:**

LINC01296 was highly expressed in clinical samples and cell lines of oral squamous cell carcinoma. Overexpression of LINC01296 promoted the proliferation, invasion, and migration of oral squamous cell carcinoma cells and accelerated the formation of xenografts, while silencing LINC01296 inhibited tumor progression. In mechanism, LINC01296 plays a tumor-promoting role by binding to SRSF1 protein.

**Conclusion:**

LINC01296 promotes malignant lesions in oral squamous cell carcinoma by binding to SRSF1 protein, which provides important experimental data and theoretical basis for the prevention, diagnosis, and treatment of oral squamous cell carcinoma.

## 1. Introduction

Head and neck cancer (HNC) is the sixth most common malignant tumor in the world. Oral squamous cell carcinoma (OSCC) is the most common subtype of head and neck cancer [1]. There are more than 500,000 new cases of oral squamous cell carcinoma in the world every year, and more than 140,000 people die of oral squamous cell carcinoma every year [2]. At present, the main methods for the treatment of OSCC are surgical resection, adjuvant radiotherapy, and chemotherapy. Although great progress has been made in surgical techniques and radiotherapy and chemotherapy, in the past 30 years, the overall survival rate of patients with OSCC has been maintained at about 50% and has not been significantly improved [3]. Therefore, in-depth study of the molecular mechanism of the occurrence and development of OSCC is the key to the development of new and effective treatments.

OSCC is characterized by strong local invasion and cervical lymph node metastasis, its occurrence and development is a complex process, by many factors, a variety of genes participate and play a synergistic role, and different genes play different roles. Previous studies have focused on OSCC protein-coding genes. With the deepening of the research, cancer researchers have found that noncoding RNA (ncRNA), especially long noncoding RNA (lncRNA), plays a crucial role in the occurrence and development of many malignant tumors [4]. However, there are few studies on the mechanism of lncRNA in the pathogenesis of OSCC.

lncRNA, whose length exceeds 200 nt, barely codes proteins. lncRNA regulates the expression of many genes at the transcriptional, posttranscriptional, and translation levels, affecting the pathological processes of many cells [5]. Current studies have shown that lncRNA is involved in many different types of biological processes, involving cell proliferation and apoptosis, body growth and development, inflammatory response, and the occurrence and development of multiple diseases, including cancer. It can be said that lncRNA regulates the occurrence and development of tumors and has long been recognized as a new choice for targeted therapy of cancer drugs. For example, studies have found that lncRNA DQ786243 downregulated in digestive tract tumors and colorectal cancer, thus weakening the proliferation and invasion of cancer cells in vitro, and weakening the growth of tumor cells in vivo [6]. In osteosarcoma, lncRNA LINC01296 regulated cyclinD1 to increase cancer cell proliferation, metastasis, and cycle [7]. In addition, it was also found that the level of lncRNA UCA1 was related to the invasiveness of bladder cancer [8]. lncRNA MALAT1 facilitated the growth of tongue cancer by targeting JAG1 [9]. Therefore, further discussion on the expression of lncRNA in OSCC is expected to provide important experimental data and theoretical basis for the prevention and treatment of OSCC.

Splicing factor SRSF1 is an important member of the serine-arginine protein family. SRSF1 has been reported to be involved in a variety of biological functions, such as translation control, meaningless mediation of RNA decay (NMD), RNA transport, and aging [10, 11]. SRSF1 has been reported as an oncogene in several cancers [12, 13]. The high expression of SRSF1 and MYC contributes to the transformation of mammary epithelial cells [14]. In addition, SRSF1 promoted the transformation of epithelial to mesenchymal (EMT) by regulating the splicing of oncogene RON [15] and controlled the alternative splicing of PRRC2C and MKNK2 to regulate the progression of lung cancer.

In the current study, we demonstrated that LINC01296, which is highly expressed in head and neck cancer, significantly induces proliferation and invasion of OSCC cells. In terms of mechanism, LINC01296 binds and regulates SRSF1 to promote the malignant degree of OSCC.

## 2. Material Method

### 2.1. Tissue Specimen

There are surgical specimens of 58 patients with OSCC tissues and adjacent normal tissues from our hospital, which were used for follow-up experimental detection. The experiment was permitted by the Ethics Review Committee of Jinan Stomatological Hospital, and the patients signed informed consent.

### 2.2. Animals

Animal experiments were permitted by the Animal Protection and Ethics Committee of Jinan Stomatological Hospital. BALB/c nude mice (female, 8-week-old) were purchased from Beijing Weitong Lihua Experimental Animal Technology Co., Ltd. (Beijing, China). There were four groups, and each group had six mice. For the experiment of xenograft, CAL-27 cells (5 × 10^6^) were suspended in 200 *μ*L normal saline and injected subcutaneously. Tumor volume was calculated according to the following formula: tumor volume (mm^3^): V (Mm^3^) = S2 (Mm^2^) × L (Mm)/2.

### 2.3. Cell Culture

All OSCC cell lines and stable-transfected CAL-27/SCC-9 with LINC01296 were purchased from CHI Scientific, Inc. (Jiangsu, China). The cells were cultured with complete medium including 89% 1640 and 10% FBS; both were purchased from Biological Industries (Beit-Haemek, Israel) and maintained in an incubator with 37°C and 5% of CO_2_ saturated humidity.

### 2.4. Colony Formation Experiment

The cell suspension was inoculated into a petri dish with 300 cells per dish. After the cells were evenly distributed, they were cultured in a cell incubator for 14~21 days. When a clone is visible to see, the culture is terminated. The medium was abandoned, and 4% paraformaldehyde was added to fix the cells for 15 min. After abandoning the fixed solution, Giemsa was added to dye for 15 min. Rinse with running water and dry. The number of effective cloned cells was observed under a microscope.

### 2.5. qRT-PCR

RNA extraction was performed using trizol reagent. NanoDrop 8000 (Thermo Scientific, Waltham, MA, USA) was used to detect the concentration and purity of RNA. The single-stranded cDNAs were synthesized from 1 *μ*g of RNA. The expression of mRNAs and miRNAs was quantified by RT-PCR with SYBR Green I (Thermo Fisher Scientific, Inc.).

### 2.6. Immunohistochemical Staining

Tumor and adjacent tissues were fixed in 4% paraformaldehyde, dehydrated, paraffin-embedded, and cut into sections. Paraffin sections of carcinoma were dewaxing to water in xylene and descending series of ethanol. Consecutive 4 *μ*m thick sections were analyzed using primary antibodies against Ki-67 (1 : 50, ProteinTech) and a HRP-conjugated goat anti-rabbit polyclonal antibody (1 : 50; Abcam) as the secondary antibody. The sections were photographed by light scope under an IX73 fluorescence microscope (Olympus, Valley, PA) and analyzed by Image J software.

### 2.7. Transfection

The OSCC cells were plated until the cell density reached 80% confluency of dishes to transfect. Plasmid or small interfering RNA (si-RNA) of SRSF1 and LINC01296 smart silencer was constructed by GeneChem (Shanghai, China). The plasmids transfected with Lipofectamine 2000 (Invitrogen, Carlsbad, CA).

### 2.8. Western Blot

After RIPA cleavage, we extracted the total protein and measured it with the BCA method. After quantitative denaturation, protein electrophoresis membrane transferred and blocked. The first incubation and second incubation were carried out according to the operation steps. The expression of the protein was expressed by the gray value. The first antibody of E-cadherin (1 : 200), vimentin (1 : 2000), and N-cadherin (1 : 500) was bought from ProteinTech (Wuhan, China).

### 2.9. Fluorescence In Situ Hybridization (FISH) Assay

The fluorescence-labeled probes of LINC01296, 18s rRNA, and U6 RNA were designed and synthesized; Ribo fluorescence was used for the FISH in situ hybridization kit (RiboBio, Guangzhou, China). The nucleus was stained with DAPI. The pictures were observed and pictures were taken with an Olympus fluorescence microscope.

### 2.10. Biotin-RNA Pull-Down

First, RNA was pretreated to form a secondary structure, and then, total cellular proteins were extracted. The total protein was first preincubated with 60 *μ*L streptavidin coated with lytic solution, and the lipopolysaccharide column (streptavidin beads) was rotated slowly at room temperature for 1 h to eliminate the background of binding to beads in the total protein. RNA was incubated with cell lysate for 1 hour and then incubated with beads coated with streptavidin. After washing the beads, add 1x protein sample buffer 30 *μ*L to the washed beads, mix them repeatedly, and heat them in boiling water for 10 min. After the sample passed western blot at 10% SDS-PAGE electrophoresis, the SRSF1 target band was detected.

### 2.11. MTT Assay

OSCC cells were plated in 96-well plates, and we used MTT assay to detect the cell viability. After transfection, MTT (0.5 mg/mL; Beyotime Biotechnology, China) was added and incubated for 3 h at 37°C. And then, 150 *μ*L of DMSO was added and incubated for 15 min. We measured the absorbance at 490 nm.

### 2.12. Transwell Assay

(24-) well transwell (Corning, USA) with or without matrigel were used to investigated cell invasion. 2 × 10^5^ 769-P or ORSC-2 were seeded on insert precoated with 1 *μ*g/*μ*L matrigel (BD Biosciences, USA). Medium including FBS was used to stimulate invasion in the bottom of wells. After 48 h, the invasion cells were stained with a 0.1% crystal violet.

### 2.13. RNA Immunoprecipitation (RIP)

The Magna RIP Kit (Millipore) was used for RIP assay according to the manufacturer's instructions. After cell lysis with RIP lysis buffer, 100 *μ*L of the lysate was incubated with RIP buffer containing magnetic beads, which were conjugated with human anti-SRSF1 and normal rabbit IgG (Santa Cruz Biotechnology). Finally, the target RNA was extracted and purified for further study by qRT-PCR assays.

### 2.14. Statistical Analysis

Data were shown as mean ± SD. Student's *t*-test or one-way ANOVA was used to compare the groups. *p* < 0.05 was considered significant.

## 3. Results

### 3.1. LINC01296 Is Highly Expressed in OSCC Tissues and Cell Lines

**Figure 1:**
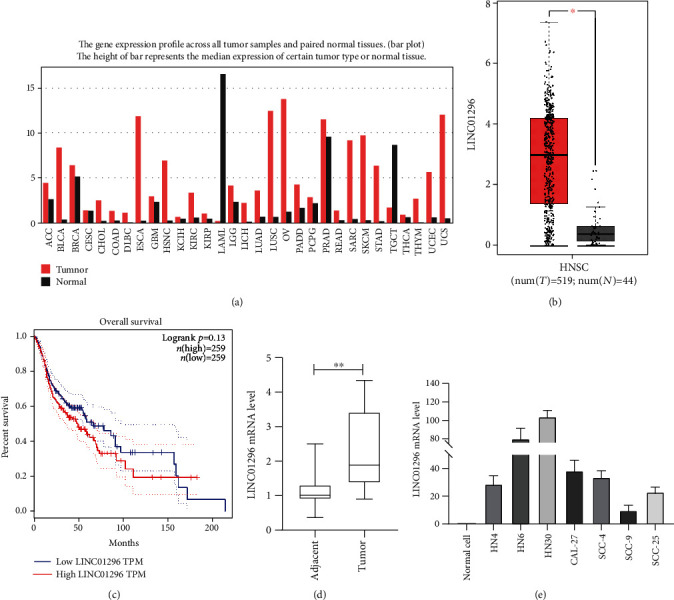
The LINC01296 expression in OSCC tissues and cell lines. (a) The LINC01296 expression in tumor samples and paired normal tissues. (b) According to the GEPIA database, LINC01296 was highly expressed in HNSC. Tumor: *n* = 519; normal: *n* = 44. (c) LINC01296 expression and patient survival analysis. (d) The level of LINC01296 in 58 cases of matched oral squamous cell carcinoma and paracancerous tissues. *N* = 58. (e) The relative expression of LINC01296 in 7 OSCC cell lines and normal cells (oral epithelial cells) was detected by qRT-PCR. *n* = 6. ^∗^*p* < 0.05; ^∗∗^*p* < 0.01.

GEPIA is a web server for cancer and normal gene expression profiling and interaction analysis based on TCGA and GTEx data, which is available at http://gepia.cancer-pku.cn/ [16]. Here, we used GEPIA to analyze differentially expressed genes found in OSCC, and we found LINC01296 is highly expressed in several types of tumors (Figure 1(a)) and significantly increased in head and neck squamous cell carcinoma (Figure 1(b)), and survival curve shows that patients with high expression of LINC01296 have a lower survival rate in the first 150 months (Figure 1(c)). We examined the carcinomatous tissues and paracancerous tissues of 58 patients with OSCC by PCR and found that LINC01296 was significantly increased in OSCC tissues (Figure 1(d)). However, it cannot be confirmed that the expression of LINC01296 is positively correlated with the pathological differentiation and lymph node metastasis of the tumor (Table 1). By comparing normal cells and 6 kinds of oral squamous cell carcinoma lines, the results revealed that LINC01296 was highly expressed in OSCC cell lines (Figure 1(e)). These results suggest that LINC01296 may participate in the occurrence and development of oral squamous cell carcinoma.

### 3.2. LINC01296 Promotes EMT of OSCC Cell Lines

**Figure 2:**
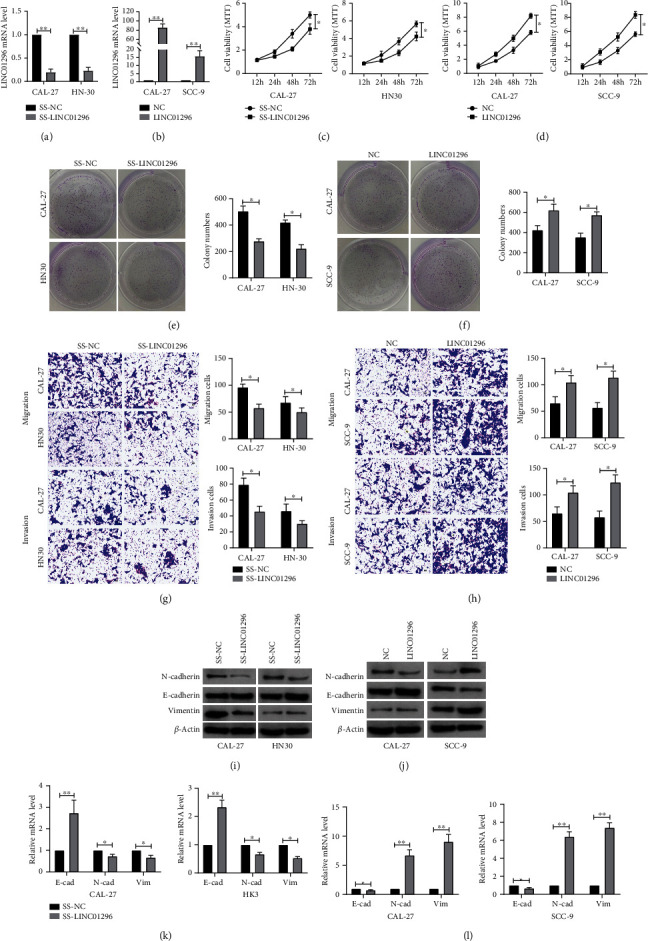
LINC01296 promotes the proliferation, migration, invasion, and EMT process of OSCC cells in vitro. (a) qRT-PCR was used to detect the relative level of LINC01296 in CAL-27 and HN30 cells 24 hours after transfection of SS-LINC01296. *n* = 6. (b) qRT-PCR was used to detect the relative stable overexpression of LINC01296 in CAL-27 and SCC-9 cells. *n* = 6. (c) The MTT method was used to detect the proliferation activity of CAL-27 and HN30 cells transfected with SS-LINC01296 and (d) CAL-27 and SCC-9 cells with LINC01296 overexpressing. *n* = 10. The colonization ability of cells transfected with SS-LINC01296 (e) and cells with LINC01296 overexpressing (f). *n* = 3. The transwell method was used to detect the migration and invasion ability of transfected with SS-LINC01296 in CAL-27 and HN30 cells (g) and CAL-27 and SCC-9 cells (h) stably overexpressing LINC0129. *n* = 3. Western blot was used to detect the E-cadherin, N-cadherin, and vimentin proteins after downregulation of LINC01296 (i) or overexpression of LINC01296 (j) in OSCC cells. *n* = 3. QRT-PCR was used to detect the E-cadherin, N-cadherin, and vimentin mRNA after downregulation of LINC01296 (k) or overexpression of LINC01296 (l) in OSCC cells. *n* = 6. NC: negative control; SS-LINC01296: LINC01296 smart silencer. ^∗^*p* < 0.05; ^∗∗^*p* < 0.01.

To explore the function of LINC01296 in OSCC, we constructed LINC01296 stable overexpression cell lines and LINC01296 smart silencer and verified their efficiency (Figures 2(a) and 2(b)). MTT assay demonstrated that knockdown LINC01296 with smart silencer inhibited the growth of OSCC cells, while overexpression of LINC01296 promoted the clone formation (Figures 2(c) and 2(d)). Through clone formation assay, we found that knockdown LINC01296 inhibited the clone formation of OSCC cells, while overexpression of LINC01296 increased the clone formation of OSCC cells (Figures 2(e) and 2(f)). Transwell assay demonstrated that knockdown of LINC01296 inhibited the migration and invasion, while overexpression of LINC01296 promoted the migration and invasion of OSCC cells (Figures 2(g) and 2(h)). As shown by Figures 2(i) and 2(k), at protein and mRNA levels, after silencing LINC01296, the level of epithelial phenotype-related marker E-cadherin was increased, while the expression of interstitial phenotype-related marker N-cadherin and vimentin was decreased. After forced expression of LINC01296, the E-cadherin was decreased and the level of N-cadherin and vimentin was increased (Figures 2(j) and 2(l)). It is suggested that high expression of LINC01296 promotes OSCC cell lines EMT.

### 3.3. LINC01296 Promotes OSCC Cell Growth In Vivo

**Figure 3:**
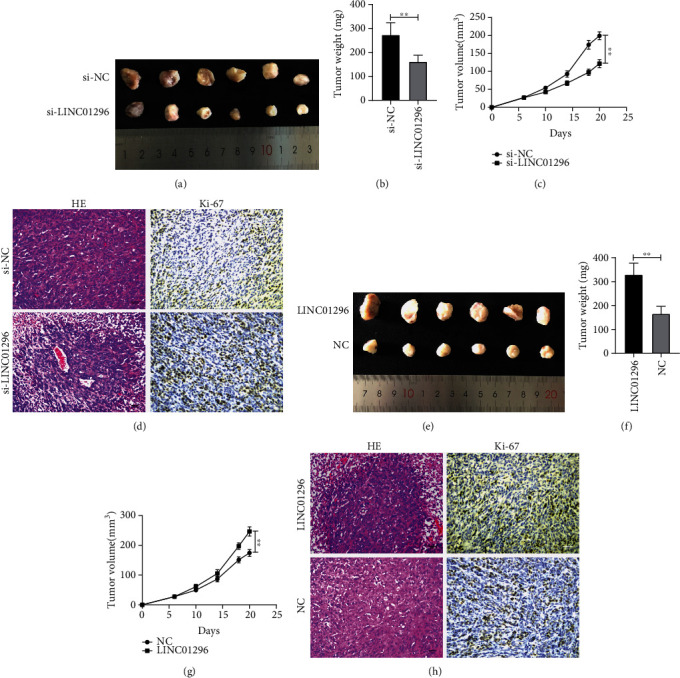
LINC01296 promotes the growth of OSCC cells in vivo. (a) Cholesterol conjugate si-LINC01296 or si-NC treated CAL-27 tumor in nude mice. The (b) weight and (c) volume of subcutaneous tumors were measured; *n* = 6. (d) Tumor tissue was stained with H&E and Ki-67 by immunohistochemical staining. *n* = 6. (e) Nude mice were subcutaneously inoculated with stable overexpressed LINC01296CAL-27 cells. *n* = 6. (f) Tumor weight and (g) volume were measured. *n* = 6. (h) The H&E and Ki-67 in tumor tissue were detected. *n* = 6. The tumor volume was calculated every 3 days for 3 weeks, and the tumor growth curve was drawn. ^∗∗^*p* < 0.01.

We used si-LINC01296-1 to carry out in vitro treatment experiment. Compared with the control group, the downregulation of LINC01296 by siRNA significantly decreased tumor growth (Figure 3(a)), which showed a decrease in tumor volume and weight in the downregulation group (Figures 3(b) and 3(c)). The changes of tumorigenesis were further confirmed by HE staining and Ki-67 staining (Figure 3(d)). Nude mice were subcutaneously inoculated with stable overexpressed LINC01296CAL-27 cells. *n* = 6 (Figure 3(e)). To the contrary, larger tumor volume and weight were observed in mice with CAL-27 cells that stably overexpressed the LINC01296 (Figures 3(f) and 3(g)), and the changes of tumorigenesis were further confirmed by HE and Ki-67 staining (Figure 3(h)).

### 3.4. LINC01296 Binds to SRSF1 Protein in OSCC Cells

**Figure 4:**
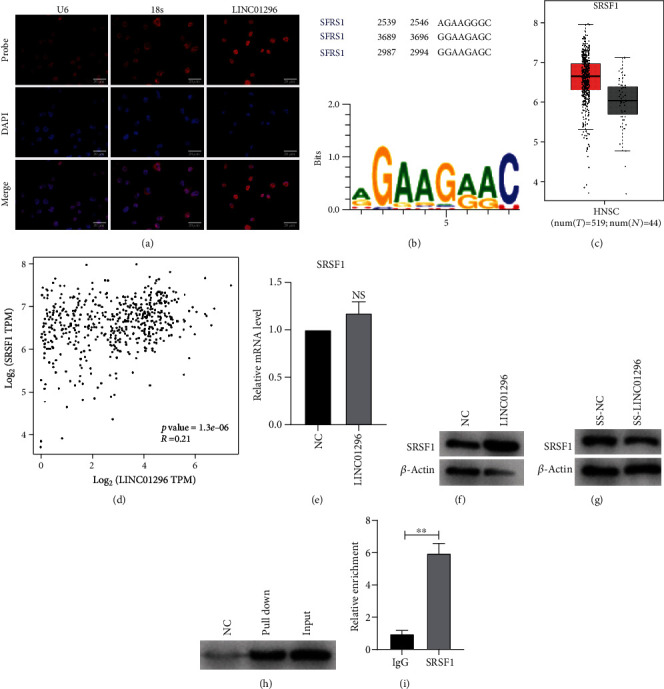
Physical interaction between SRSF1 and LINC01296. (a) The localization of LINC01296 in CAL-27 cells was analyzed by FISH. The nucleus was stained with DAPI, and the cytoplasm was labeled with 18s. (b) Binding sites and motifs were predicted. (c) Expression of SRSF1 in head and neck cancer was predicted by GEPIA. (d) Correlation between LINC01296 and SRSF1 expression in head and neck cancer. (e) The PCR method was used to detect the effect of LINC01296 on SRSF1 mRNA. *n* = 6. (f) The effect of LINC01296 overexpression on the SRSF1 protein level was measured by western blot. *n* = 3. (g) The effect of silencing LINC01296 on the level of SRSF1 protein was detected by western blot. *n* = 3. (h) The binding relationship between LINC01296 and SRSF1 protein was verified by RNA pull-down assay. (i) The RIP experiment further proved the binding relationship between LINC01296 and SRSF1. ^∗∗^*p* < 0.01; *NS*: no significant.

To clarify the mechanism of LINC01296, we first detected the localization of LINC01296 in cells by FISH experiment. The results showed that LINC01296 was more expressed in the nucleus of CAL-27 (Figure 4(a)). We speculated that LINC01296 may play a role in binding proteins in the nucleus. We use RBPDB (http://rbpdb. http://ccbr.utoronto.ca/) [17] to predict the proteins that may bind to it; the results suggest that the oncogene SRSF1 may bind to LINC01296 and predict the possible motif and their binding site (Figure 4(b)). The results of GEPAI database analysis showed that SRSF1 was high expression in head and neck squamous cell carcinoma (Figure 4(c)) and positively correlated with the expression of LINC01296 (Figure 4(d)). PCR assay showed that LINC01296 did not affect the SRSF1 mRNA expression (Figure 4(e)) but promoted the expression of SRSF1 protein (Figure 4(f)). At the same time, silencing LINC01296 can reduce the protein level (Figure 4(g)) of SRSF1. The RNA pull-down and RIP result of Figures 4(h) and 4(i) revealed the binding relationship between LINC01296 and SRSF1.

### 3.5. SRSF1 Facilitates EMT of Oral Squamous Cell Carcinoma Cell Line

**Figure 5:**
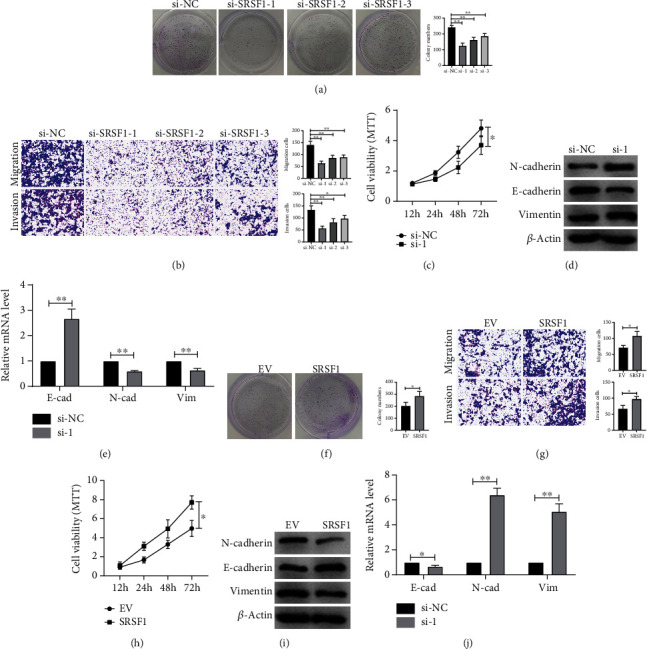
SRSF1 promotes proliferation, invasion, and EMT of OSCC cell line. The colony formation ability of the cells transfected with (a) si-SRSF1 or (f) SRSF1 plasmid was detected. *n* = 3. The migration ability of cells transfected with (b) si-SRSF1 or (g) SRSF1 plasmid was detected by the transwell method. *n* = 3. (c) si-SRSF1 or (h) SRSF1 plasmid was transfected into CAL-27 cells; viability was detected by the MTT method. *n* = 10. E-cadherin, N-cadherin, and vimentin protein levels in CAL-27 cells when SRSF1 was knocked down (d) or overexpressed (i). *n* = 3. The mRNA level of markers E-cadherin, N-cadherin, and vimentin in CAL-27 cells when SRSF1 was knocked down (e) or overexpressed (j). *n* = 6. ^∗^*p* < 0.05; ^∗∗^*p* < 0.01.

SRSF1 has been reported as an oncogene in many types of tumors, but its role in OSCC has not been reported. In order to explore the function of SRSF1 in oral squamous cell carcinoma, we constructed SRSF1 overexpression plasmid and siRNA. Through clone formation assay, we found that knockdown SRSF1 inhibited the growth of OSCC cells, while overexpression of SRSF1 facilitated the clone formation of OSCC cells (Figures 5(a) and 5(f)). Transwell assay showed that knockdown of SRSF1 weakens the migration and invasion ability of OSCC cells, while overexpression of SRSF1 accelerated the malignant behavior (Figures 5(b) and 5(g)). MTT assay showed that knockdown of SRSF1 inhibited the cell vitality of OSCC cells, while overexpression of SRSF1 promoted the cell vitality (Figures 5(c) and 5(h)). As shown by Figures 5(d) and 5(i) and Figures 5(e) and 5(g), at protein and mRNA levels, after silencing SRSF1, the level of epithelial phenotype-related marker E-cadherin was upregulated, while the expression of interstitial phenotype-related marker N-cadherin and vimentin was inhibited. After forced expression of SRSF1, E-cadherin expression was decreased and the expression of N-cadherin and vimentin was increased (Figure 5(j)). It is suggested that high expression of SRSF1 promotes OSCC cell lines EMT.

### 3.6. SRSF1 Mediates the Effect of LINC01296 to Promote the EMT of OSCC Cells

**Figure 6:**
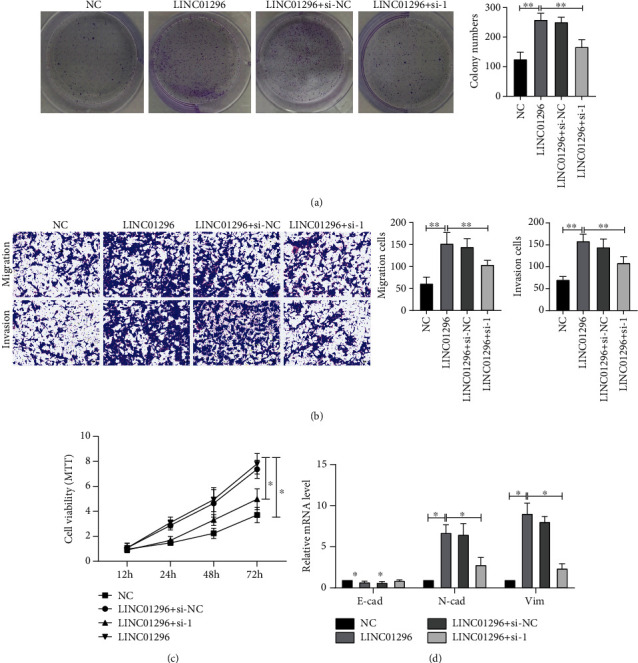
SRSF1 mediates the effect of LINC01296 on EMT of OSCC cells. (a) Clone formation assay was used to detect the clone formation ability of silenced SRSF1 in CAL-27 cells with stable overexpression of LINC01296. *n* = 3. (b) The effect of silencing SRSF1 on the migration and invasion ability of stably overexpressed LINC01296 CAL-27 cells was measured by the transwell method. *n* = 3. (c) The proliferation ability was detected in stably overexpressed LINC01296 CAL-27 cells when SRSF1 was knocked down. *n* = 10. (d) Using RT-PCR to detect the mRNA level of E-cadherin, N-cadherin, and vimentin in LINC01296 stably overexpressed CAL-27 cells when SRSF1 was knocked down. *n* = 6. ^∗^*p* < 0.05; ^∗∗^*p* < 0.01.

In order to further determine that SRSF1 participates in the function of LINC01296, we used SRSF1 siRNA-1 while overexpressing LINC01296. Colony formation assay, transwell, and MTT assay showed that silencing SRSF1 reversed the proliferation, migration, and invasion of CAL-27 induced by high expression of LINC01296 (Figures 6(a)–6(c)). PCR results showed that inhibition of SRSF1 expression reversed the epithelial-mesenchymal transformation (Figure 6(d)) of CAL-27 cells induced by LINC01296, which proved that SRSF1 mediated the function of LINC01296.

## 4. Discussion

Less than 2% of the RNAs transcribed from the human genome sequence can encode proteins, and about 98% of the RNAs that cannot encode proteins are called noncoding RNA [18, 19]. More than 68% of the genes belong to lncRNA [20]. In the past, lncRNA was regarded as nonfunctional “noise,” but in recent years, many researches have demonstrated that the abnormal expression of lncRNA is closely linked to the tumorigenesis and development and is an important potential therapeutic target and diagnostic and prognostic marker of cancer [21, 22].

lncRNA has the characteristics of strong tissue specificity, and its expression and function may be very different in diverse cells [23]. Recently, it has been found that LINC01296 is highly expressed in kinds of cancers, including ovarian cancer, pancreatic ductal carcinoma, and hepatocellular carcinoma. The high expression of LINC01296 is significantly related to tumor size, clinical stage, and survival time of patients and plays an important role in promoting cancer in many cancers [24–26]. Feng et al. [27] transcript analysis showed that LINC01296 was highly expressed in OSCC compared with normal samples, but the function and mechanism of LINC01296 in OSCC was not clear. In this study, through database analysis, it was also found that LINC01296 was higher expression in head and neck squamous cell carcinoma, which may relate to the survival of patients; only in the first 150 months, high expression patients have a lower survival rate. However, there was no statistical significance between overall survival and the expression of LINC01296, and more clinical data should be included to verify the relationship between the LINC01296 level and survival rates. These results suggest that LINC01296 may be a crucial therapeutic and prognostic target in the early stage of patients, so the in-depth study of LINC01296 has an extensive and important prospect of clinical transformation.

In this study, qRT-PCR was used to detect 58 of OSCC and paracancerous tissues. The results showed that the expression of LINC01296 in oral squamous cell carcinoma was significantly higher than that in paracancerous tissues. Furthermore, qRT-PCR was used to determine the expression of LINC01296 in normal oral mucosal cells and OSCC cell lines. The results suggested that the expression of LINC01296 in OSCC cell lines was significantly higher than that in normal oral mucosal cells. Therefore, we have proved that the expression of LINC01296 is increased in OSCC from the level of clinical specimens and cell lines in vitro.

The changes of cell proliferation, invasion, and metastasis are important reasons for the tumorigenesis and development of cancer. By detecting cell proliferation, migration, invasion, and EMT-related markers after interfering with the expression of LINC01296 in oral squamous cell carcinoma cell lines, it was proved that silencing LINC01296 in OSCC cells could inhibit cell proliferation and inhibit cell EMT. Overexpression of LINC01296 can promote the malignant degree of OSCC. Furthermore, through database analysis, RNA pull-down test, and cell level recovery test, it is proved that LINC01296 promotes EMT and tumorigenesis by binding to SRSF1. However, due to the nonspecificity of LINC01296 in combination with SRSF1, LINC01296 may regulate other downstream factors, which need to be further explored.

Malakar et al. [28] found that lncRNA MALAT1 regulates the alternative splicing of oncogenes by upregulating SRSF1 and plays a tumor-promoting role as a protooncogene in hepatocellular carcinoma. Fu et al. [29] have shown that the overexpression of SRSF1 and SRSF9 promotes the recruitment of *β*-catenin mRNA to promote tumorigenesis. In future experiments, we will further explore the mechanism of SRSF1 in OSCC and improve the regulatory pathway of LINC01296-SRSF1 in OSCC.

## Figures and Tables

**Table 1:** Clinicopathologic features of patients (*N* = 58).

Characteristics	No. of patients	No. %
Age (years)		
≥60	37	63.79
<60	21	36.21
Gender		
Male	42	72.41
Female	16	27.59
Tumor size (cm)		
≤4	47	81.03
>4	11	18.97
TNM stage		
I	23	39.66
II	21	36.21
III	9	15.51
IV	5	8.62
Pathological		
Differentiation		
Well	40	68.97
Moderately/poorly	18	31.03
Local invasion		
No	41	70.69
Yes	17	29.31

## Data Availability

The datasets are available from the corresponding author upon reasonable request.
